# Cultural adaptation and psychometric characteristics of the child oral and motor proficiency scale (ChOMPS)–Turkish version

**DOI:** 10.55730/1300-0144.6069

**Published:** 2025-07-13

**Authors:** Ebru UMAY, Sibel EYİGÖR, Damla CANKURTARAN, Sema KALKAN, Nihal TEZEL, Cuma UZ, Şükran GÜZEL, Fatma BALLI UZ, Güler GÖZPINAR, Recep GAYIR, Fatma NAZLI, Kerim DEMİRSÖZ, Ece ÜNLÜ AKYÜZ, Britt PADOS

**Affiliations:** 1 Department of Physical Medicine and Rehabilitation, Etlik Physical Medicine and Rehabilitation City Hospital, Ankara, Turkiye; 2 Department of Physical Medicine and Rehabilitation, Ege University Faculty of Medicine, İzmir, Turkiye; 3 Department of Pediatric Metabolism, Ege University Faculty of Medicine, İzmir, Turkiye; 4 Department of Physical Medicine and Rehabilitation, Erdemli State Hospital, Mersin, Turkiye; 5 Department of Physical Medicine and Rehabilitation, Kırklareli University Faculty of Medicine, Kırklareli, Turkiye; 6 Infant Feeding Care, Wellesley, Massachusetts, Unıted States of America

**Keywords:** Deglutition, children, ChOMPS, validity, reliability

## Abstract

**Background/aim:**

The child oral and motor proficiency scale (ChOMPS) is a parent-reported assessment that evaluates a child’s eating and drinking skills within the framework of all related motor functions. It has been found to be useful in research studies and recommended in reviews. The purpose of this study was to perform the translation and cultural adaptation of the English version of the ChOMPS to Turkish and conduct psychometric testing of the ChOMPS–Turkish version, including reliability and validity.

**Materials and methods:**

This study was conducted with 185 children. Cronbach’s α, corrected item-to-total correlations, coefficient of variation, and Cronbach’s α when one item was deleted were used to assess internal consistency. In addition, test–retest reliability was assessed. The functional oral intake scale (FOIS) and pediatric eating assessment tool-10 (Pedi-EAT-10) scales were used for convergent validity. Moreover, Pedi-EAT-10 score was used to perform the receiver operating characteristic (ROC) curve analysis and the sensitivity and specificity of ChOMPS–Turkish version were calculated.

**Results:**

It was found that the ChOMPS–Turkish version demonstrated acceptable validity and reliability. Cronbach’s α levels were excellent (0.969 and 0.973), and test–retest reliability demonstrated very high agreement (0.997–0.999; p < 0.001). Significant good-to-excellent correlations were found between the validation scales. In addition, the total ChOMPS–Turkish version score for dysphagia risk as estimated using Pedi-EAT-10 had 94.74% sensitivity and 80.28% specificity.

**Conclusion:**

The ChOMPS–Turkish version demonstrates strong evidence of validity and reliability for use in clinical practice and research.

## 1. Introduction

Feeding difficulties are common in young children [1] and can impact nutrition, hydration, growth, and development [2]. Feeding is highly complex and difficulties can arise from a variety of underlying issues. Children may have difficulty with oral-motor functioning, which impacts their ability to appropriately move food in the mouth, keep it in the mouth, or chew foods of more complex textures [3]. Children may also have difficulties with swallowing (i.e. dysphagia) that impacts their ability to safely and effectively move food from the mouth to the stomach while protecting the airway [4]. They may have difficulty maintaining physiologic stability and endurance to eat and drink enough to be adequately hydrated and nourished. Feeding also requires gross and fine motor movements to move food to the mouth and hold the body in a position for safe and effective swallowing. Sensory and cognitive differences can have a significant impact on all of these areas of oral feeding.

Feeding and swallowing disorders are more common in children with medical complexity and a history of prematurity [5–9], but they can also be seen in 25%–45% of children with normal development [2]. In children with medical complexity, complications related to feeding difficulties can contribute to increased morbidity and mortality [1]. Early diagnosis and treatment are important for optimal health outcomes. Screening tests can help to facilitate early identification and diagnosis of pediatric feeding disorder [10–14]. Parent-reported scales allow for an inexpensive, easy-to-implement, and noninvasive measurement that can be implemented quickly when a problem is suspected [15].

The child oral and motor proficiency scale (ChOMPS) is a parent-reported assessment that evaluates a child’s eating and drinking skills within the framework of all related motor functions [3]. The ChOMPS, in its original English version, has been shown to be a valid and reliable scale [16–17], and it has been found to be useful in research studies [18–19] and recommended in reviews [20–21]. The purpose of this study was to perform the translation and cultural adaptation of the English version of the ChOMPS to Turkish and conduct psychometric testing of the ChOMPS–Turkish version, including reliability and validity.

## 2. Materials and Methods

This study was approved by the local Institutional Ethics Committee (P202200016/03) and carried out in a pediatric rehabilitation outpatient clinic between November 2022 and May 2023. All procedures were conducted in accordance with the relevant principles of the 2004 Helsinki Declaration. Children aged 6 months to 7 years, with and without swallowing problems, who had a regular caregiver with cognitive and linguistic skills sufficient to understand the questions were eligible for inclusion in the study. Children with medical instability, such as an acute infection that may affect the retest results or medication adjustment that may affect swallowing function, were excluded. Before participation in the study, the caregivers of eligible children (legal guardians) were given verbal and written information on the nature of the study. Informed consent forms were signed upon admission to the trial.

At the beginning of the study, demographic characteristics were obtained from the parents, and a general physical examination of the children was performed. Demographic characteristics such as age, sex, body mass index, disease, and swallowing-related characteristics such as independent feeding status, diet, and feeding method modification were recorded.

After examination of all children, the FOIS scale was completed by the examining physician. The Pedi-EAT-10 and ChOMPs scales were completed by questioning the parents.

Functional oral intake scale (FOIS) is a physician-reported classification of the swallowing functions of children [22]. FOIS is a scale that classifies the oral intake status of children into seven categories, from the first level “nothing by mouth” to the seventh level “total oral intake, no restrictions”. The first three levels indicate tube-dependent enteral feeding status, while levels four through seven indicate various degrees of oral dietary modifications.

The pediatric eating assessment tool-10 (Pedi-EAT-10) is a ten-item, parent-reported instrument that evaluates dysphagia symptoms and severity [23]. Each question is scored from 0 (no problem) to 4 (severe problem), and children whose total score is ≥ 4 are considered to have an elevated dysphagia risk. The Pedi-EAT-10 has been found to have acceptable specificity and sensitivity [24].

The child oral and motor proficiency scale (ChOMPS) is a scale that provides a measurement of eating, drinking, and related motor skills based on parent report in infants and young children aged 6 months to 7 years [3]. The ChOMPS was developed to measure observable eating skills (what a child can and cannot do). The ChOMPS consists of a total of 63 questions, including complex movement patterns (23 questions), basic movement patterns (20 questions), oral-motor coordination (14 questions), and fundamental oral-motor skills (six questions) [17]. Each question begins with the phrase “My child can ___” and response options are on a three-point scale: the skill can be consistently completed (yes = 2 points), the skill can sometimes be completed (sometimes = 1 point), and the skill cannot yet be consistently achieved (not yet = 0 points). Higher scores indicate greater levels of skill, and the total possible range of scores is 0 to 126. Reference values are available for 11 different age groups between 6 months and 7 years and can be used to determine whether a score is within the range of typical for the child’s age [16]

**Figure 1:**
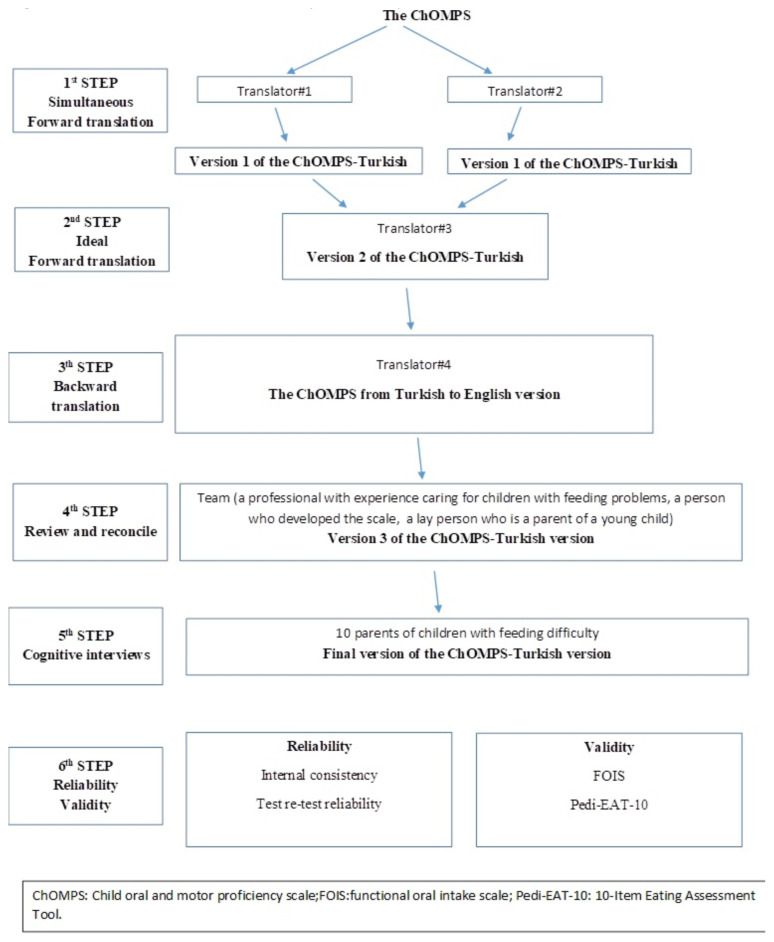
Translation and cultural adaptation steps of the ChOMPS.

Permission to use and translate the questionnaire was obtained from the original author (BF. Pados) [3]. Translation and cultural adaptation were done in steps 1–5, and reliability and validity testing was completed in step 6 (Figure 1). In step 1, a simultaneous and independent forward translation (i.e. translation from English to Turkish) was conducted by two native Turkish speakers. In step 2, a third native speaker reviewed the two translations created in step 1, reconciled the differences, and decided on the ideal forward translation.

In step 3, a fourth native speaker (who had not been involved before and had not seen the original tool) translated the Turkish version created in step 2 back into English. In step 4, a team was formed to review and troubleshoot the English version created in step 3 by comparing it with the original tool. This team included one of the original authors of the English version of the ChOMPS, a professional with experience working with children with feeding and swallowing problems, and a layperson who was the Turkish-speaking parent of a young child. The scale was finalized by this team. In step 5, cognitive interviews were conducted with 10 Turkish-speaking parents of children with feeding difficulties to ensure that the language used was understandable to the parents. The final version was created with their feedback. In step 6, psychometric tests were performed to assess the validity and reliability of the scale.

Internal consistency reliability was measured using Cronbach’s α, corrected item-to-total correlations (Spearman rank correlation coefficient test), coefficient of variation, and Cronbach’s α when one item was deleted. A Cronbach’s α of 0.70 or higher and an item-to-total correlation of 0.30 and/or higher were considered to indicate adequate reliability [25].

To evaluate temporal stability, test–retest reliability was calculated using Cohen’s κ coefficients for individual items between ChOMPS scores collected 2 weeks apart. A κ coefficient of 0–0.40 was considered poor, 0.41–0.60 fair to good, and 0.61–1.00 excellent [26]. In addition, the intraclass correlation coefficient (ICC) for the total ChOMPS and subscale scores was calculated for test–retest reliability. According to the ICC results, positive values ranging from 0 to 0.20 indicate poor agreement; 0.21 to 0.40 indicate fair agreement; 0.41 to 0.60 indicate moderate agreement; 0.61 to 0.80 indicate good agreement; and 0.81 to 1.00 indicate very good agreement [27].

The FOIS and Pedi-EAT-10 scales were used for convergent validity. The Spearman correlation test was used to assess the association between Pedi-EAT-10 and FOIS, as well as the total and subscale scores of ChOMPS. The correlation coefficient (r) was used to indicate the strength of the correlation. Accordingly, r < 0.30 indicated a weak correlation; r = 0.30–0.50 indicated a moderate correlation; r = 0.51–0.75 indicated a good correlation; and r = 0.76–1.00 indicated a very good correlation between the variables [27].

Additionally, the sensitivity and specificity of the ChOMPS–Turkish version were evaluated. Total ChOMPS scores were interpreted according to reference values determined according to age [14], and children were determined to have either normal feeding skills (i.e. score ≥ 90th percentile for age) or abnormal feeding skills (scores < 90th percentile for age). According to the Pedi-EAT-10 scores, children were grouped as either having dysphagia (score ≥ 4) or not having dysphagia (score < 4). Receiver operating characteristic (ROC) curves were generated to analyze the correlation between the total ChOMPS–Turkish version score and the Pedi-EAT-10. The area under the curve (AUC), sensitivity and specificity values, positive and negative likelihood ratios (LR), positive and negative predictive values (PV), and accuracy were calculated [28].

All statistical analyses were carried out using R software version 4.0 (The R Foundation for Statistical Computing, Vienna, Austria) and the SPSS 25.0 statistical package (International Business Machines-IBM Corporate, Armonk, New York, USA). Continuous variables were analyzed using the Kolmogorov–Smirnov test to assess whether they were normally distributed. Descriptive statistics were demonstrated as mean ± standard deviation (SD) and median (minimum–maximum) for continuous variables and as a percentage (%) for nominal variables. The percentage of missing data and the percentage of computable points were determined to assess data quality. Floor and ceiling effects were evaluated to determine acceptability. Floor or ceiling effects less than 15% were defined as acceptable. For all tests, p < 0.05 was considered statistically significant.

## 3. Results

A total of 192 children followed at the outpatient clinic were initially included in the study. Seven children participated in the initial assessment but did not participate in the retest evaluation and were therefore excluded from the study. The study was conducted with 185 children.

The mean age of the children was 57.54 ± 23.66 months (range: 8.0–84.0 months), and the mean body mass index (BMI) was 20.22 ± 4.80. The majority of the children (n = 163, 88.1%) had cerebral palsy, and none of them had typical development (n = 0). Demographic characteristics of the participants are shown in Table 1. The mean FOIS score for the sample was 5.40 ± 0.96, and the median Pedi-EAT-10 score was 4 (range: 0–36).

The application rate of the ChOMPS–Turkish version was 100%. The percentage of missing data was 0% for all items, and the percentage of computable scores was 100%. The ceiling effect was 4.9% and 7.6%, and the floor effect was 0%, all within acceptable limits.

The mean total score of the ChOMPS–Turkish version was 96.17 ± 18.35. Subscale scores were: mean 30.30 ± 7.64 for the complex movement patterns subscale, 31.58 ± 6.71 for the basic movement patterns subscale, 24.06 ± 5.75 for the oral-motor coordination subscale, and 10.75 ± 2.83 for the fundamental oral-motor skills subscale. On the 2-week retest, the mean total score of the ChOMPS–Turkish version was 94.29 ± 17.57. Retest subscale scores were 29.67 ± 7.34 for the complex movement patterns subscale, 31.16 ± 6.62 for the basic movement patterns subscale, 23.81 ± 5.65 for the oral-motor coordination subscale, and 10.72 ± 2.80 for the fundamental oral-motor skills subscale.

The internal consistency of the total ChOMPS–Turkish version at each time point (test and retest) was excellent, with Cronbach’s α values of 0.973 and 0.969, respectively. Cronbach’s α values for all subscales at both time points were also very good: complex movement patterns = 0.924 and 0.928; basic movement patterns = 0.956 and 0.953; oral-motor coordination = 0.947 and 0.940; and fundamental oral-motor skills = 0.957 and 0.955. Corrected item–total correlation coefficients ranged from 0.606 and 0.902, indicating moderate to very good agreement (Table 2). Both test and retest Cronbach’s α values for each item, when one item was deleted, were lower than the total ChOMPS–Turkish version Cronbach’s α value (ranging from 0.765 [item 27] to 0.796 [item 46] in the test, and from 0.723 [item 27] to 0.756 [item 8] in the retest). The coefficient of variation for the total ChOMPS–Turkish version was 18.7% (test) and 18.1% (retest). The coefficient of variation for individual items ranged from 10% in the test (item 35) to 11% in the retest (item 28), and from 28% in the test (item 45) to 29% in the retest (item 8), which are considered acceptable values (Table 2). Please see Supplemental Material for the items of the English translation of the ChOMPs-Turkish version.

Cohen’s κ coefficients for individual items ranged from 0.846 to 1.000 (Table 2). The ICC coefficients for both the total ChOMPS–Turkish version scores and the subscale scores indicated very good agreement (0.997–0.999; p < 0.001) (Table 3).

Significant good-to-excellent negative (Pedi-EAT-10) and positive (FOIS) correlations were found between the validation scales and both the total ChOMPS–Turkish version and its subscales (correlation coefficients between –0.602 and –0.859, and between r = 0.562 and 0.708, respectively) (Table 4).

Based on the interpretation of the total ChOMPS–Turkish version scores using age-based reference values, 65.9% (n = 122) of the children had abnormal feeding skills (i.e. a score < 90th percentile for their age), while 34.1% (n = 63) had normal feeding skills (i.e. a score ≥ 90th percentile for their age). According to the Pedi-EAT-10 scores, 114 (61.6%) of the children had difficulty swallowing, while 71 (38.4%) did not (Table 5).

**Figure 2:**
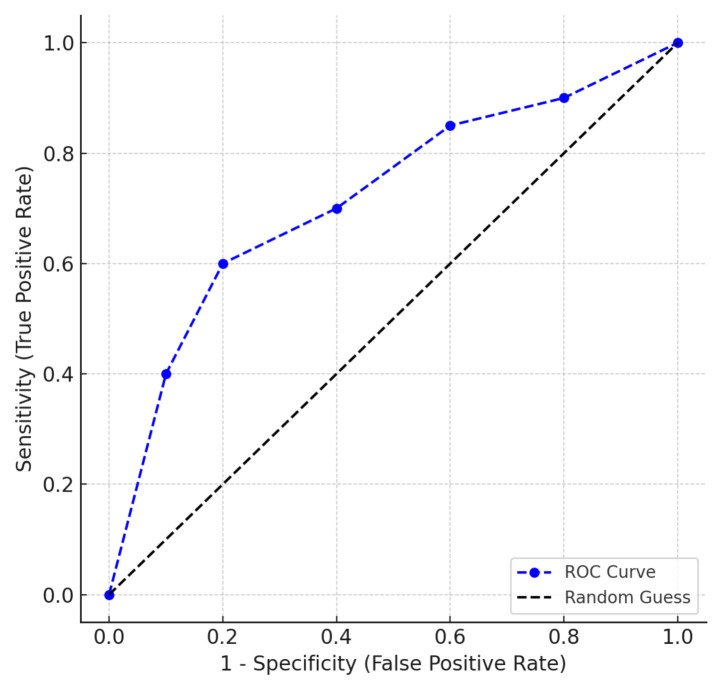
ROC curve analysis results.

The total score of the ChOMPS–Turkish version, in estimating dysphagia risk based on the Pedi-EAT-10, showed 94.74% sensitivity and 80.28% specificity (Table 6 and Figure 2). In addition, high AUC (0.875), positive PV, and LR demonstrated high sensitivity and specificity. These results indicate that the ChOMPS–Turkish version has good diagnostic accuracy for dysphagia [27].

## 4. Discussion

This study describes the translation and cultural adaptation process of the English version of the ChOMPS into Turkish and reports on the psychometric testing of the ChOMPS–Turkish version. In this sample of 185 children with neurologic conditions, the ChOMPS–Turkish version demonstrated internal consistency reliability, test–retest reliability, convergent validity, and high sensitivity and specificity. The original English version of the ChOMPS was found in a study of 364 children to have evidence of adequate internal consistency reliability (Cronbach’s α = 0.74–0.97) [17]. Our study found slightly higher Cronbach’s α (between 0.924 and 0.973), suggesting that the Turkish version continues to have excellent internal consistency reliability. Test–retest reliability of the Turkish version was higher than that reported for the original English version, likely due to the more homogeneous sample in our study (88.1% of the sample was diagnosed with cerebral palsy), compared to the highly heterogeneous sample, both in terms of age and medical diagnoses, included in the original study [17].

The ChOMPS–Turkish version was also found to have evidence of adequate convergent validity with the FOIS and Pedi-EAT-10. Convergent validity in the original English version of the ChOMPS was evaluated using the gross and fine motor developmental domains of the Ages & Stages Questionnaires—Third edition (ASQ-3) (for children aged 6 months to 5 years) and the PROMIS Pediatric Parent Proxy Short Form—Mobility and Upper Extremity (for children aged 5–7 years) [17]; however, these measures are not available in Turkish, so alternatives were required for this study.

In this study, as expected, children with better feeding skills (i.e. higher ChOMPS scores) had higher scores on the FOIS (i.e. more independent oral feeding). Children with lower feeding skill levels (i.e. lower ChOMPS scores) had higher Pedi-EAT-10 scores (i.e. greater risk of dysphagia). The development of gross motor control of the head and spine, especially in infants, is closely related to oral sensorimotor development and oral function, and the transition from liquid foods to semisolid and solid foods begins in parallel with the development of postural control [10,29]. It has been reported that the risk of aspiration increases in parallel with poor motor development and postural control, especially in children with neurological disease and oropharyngeal dysphagia [29–31], which is further supported by our study findings. The ChOMPS–Turkish version was found to have high sensitivity and specificity for identifying dysphagia.

Another important point in this study was the high median Pedi-EAT-10 score (median = 4; range 0–36). Given that a score of 4 indicates elevated risk, this median suggests that a significant proportion of the study population indeed had dysphagia symptoms. Along with our other findings, this high median value suggests that we must carefully consider the risk of dysphagia in children with neurological disorders.

One limitation of this study is the uneven age distribution among participants. The ChOMPS is a scale that allows assessment across 11 different age ranges starting from 6 months. In our study, there were no patients between 6 months and 9 months. Therefore, the ChOMPS–Turkish version can be considered applicable to children aged 9 months to 7 years. Future studies of the ChOMPS–Turkish version should explore the psychometric properties of the scale in infants as young as 6–9 months. Additionally, validation of the ChOMPS–Turkish version with clinician and/or instrumental assessments, such as videofluoroscopy or flexible fiberoptic endoscopy, could provide additional support for the use of this assessment in young children with pediatric feeding disorder.

In conclusion, the ChOMPS–Turkish version is a 63-item parent-reported measure of eating, drinking, and related skills intended for use with Turkish-speaking parents of children aged 9 months to 7 years. Similar to the original English version, the Turkish adaptation of ChOMPS demonstrates strong validity and reliability for use in clinical practice and research.

## Supplemental Material

English Translation of the Child Oral and Motor Proficiency Scale (ChOMPs)-Turkish Version

Item 1. Stand without holding on to anything.
Item 2. Walk 10–20 steps by himself/herself
Item 3. Run 10–20 steps without falling
Item 4. Climb 2–3 stairs holding on to someone or something
Item 5. Climb 2–3 stairs without holding on to someone or something
Item 6. Go down 2–3 stairs holding on to someone or something
Item 7. Go down 2–3 stairs without holding on to someone or something
Item 8. Jump with both feet without holding on to anything
Item 9. Drink from an open cup held by an adult with little or no spilling of liquid from mouth
Item 10. Hold an open cup and drink by himself/herself with little or no spilling of liquid from mouth
Item 11.Keep tongue in his/her mouth when drinking from an open cup (held by self or an adult)
Item 12. Drink from an open cup holding rim with lips (without biting rim with teeth)
Item 13. Use a filled spoon or fork to bring food to mouth
Item 14. Scoop food onto a spoon or fork and bring it to his/her mouth
Item 15. Use a fork to stab a piece of food and bring it to his/her mouth
Item 16. Use tongue to lick food off his/her top lip
Item 17. Use tongue to lick food from corners of his/her mouth
Item 18. Use upper teeth or lip to Clean food from his/her bottom lip
Item 19. Pucker his/her lip to kiss or blow
Item 20. Drink with a straw
Item 21. Take a bite of hard, crunchy foods such as carrot sticks
Item 22. Eat hard, crunchy foods such as raw carrot sticks without gaging, coughing, or choking
Item 23. Speak using words that people outside our family can understand
Item 24. Hold his/her head upright when lying on his/her tummy
Item 25. Pull up to stand
Item 26. Stand holding on to something (such as a table or couch).
Item 27. Walk holding on to someone or something
Item 28. Support his/her weight on her/his forearms when lying on his/her tummy
Item 29. Bring one or both hands to his/her mouth
Item 30. Hold a toy in hand
Item 31. Bring a toy or a piece of food to his/her mouth.
Item 32. Hold a bottle or sippy cup
Item 33. Bring a bottle or sippy cup to his/her mouth
Item 34. Roll over from his/her tummy to his/her back
Item 35. Use his/her fingers like a rake to bring food or a toy towards himself/herself
Item 36. Grasp a piece of food (such as cheerio cereal) between thumb and another finger
Item 37. Move a toy or a piece of food from one hand to the other hand
Item 38. Roll over from his/her back onto his/her tummy
Item 39. Keep his/her head steady when in a supported position (such as with back against a chair or while being held)
Item 40. Sit up with support (such as sit with back against a chair or while being held)
Item 41. Sit up straight with no support
Item 42. Twist his/her body to their left and right when sitting unsupported (such as reaching for a toy or looking at something)
Item 43. Crawl when placed on his/her tummy
Item 44.Use tongue to move food around in his/her mouth
Item 45. Keep solid food in his/her mouth while eating
Item 46. Keep tongue in his/her mouth when food is offered on a spoon
Item 47. Move his/her jaw up and down to chew
Item 48. Drink thin liquids such as water or juice without gagging, coughing, or choking
Item 49. Take a bite of soft food, such as cake or banana
Item 50. Take a bite of firm food, such as crackers or cookies
Item 51. Eat a spoonful of smooth food, such as baby formula eaten with a spoon or yoghurt, without gagging, coughing, or choking
Item 52. Eat food that dissolves, such as baby snack (puff) that dissolves in the mouth and does not require chewing, without gagging, coughing, or choking
Item 53. Eat soft food, such as a pancake, without gagging, coughing, or choking
Item 54. Eat textured food, such as cornflake with milk, without gagging, coughing, or choking
Item 55. Eat textured food with some lumps, such as lightly mashed bananas, without gagging, coughing, or choking
Item 56. Eat firm food, such as peeled apple slice, without gagging, coughing, or choking
Item 57. Eat chewable food, such as a piece of hot dog, without gagging, coughing, or choking
Item 58.Close his/her lips completely
Item 59. Move his/her tongue inside mouth from side to side
Item 60. Stick his/her tongue out past the teeth or gums
Item 61. Open his/her mouth wide enough to accept a spoon
Item 62. Bite down so that teeth or gums touch
Item 63. Move his/her chin down to his/her chest

## Figures and Tables

**Table 1:** Demographic and swallowing characteristics of the participants (n = 185).

Variables	n (%)

**Age**	
6–9 months	0
9 months 1 day–12 months	5 (2.7)
12 months 1 day–15 months	6 (3.2)
15 months 1 day–18 months	7 (3.8)
18 months 1 day–24 months	7 (3.8)
2 years 1 day–2.5 years	9 (4.9)
2,5 years 1 day–3 years	8 (4.3)
3 years 1 day–4 years	30 (16.2)
4 years 1 day–5 years	19 (10.3)
5 years 1 day–6 years	51 (27.6)
6 years 1 day–7 years	43 (23.2)

**Sex**	
Female	30 (16.2)
Male	155 (83.8)

**Disease**	
Cerebral palsy	163 (88.1)
Neuromuscular disease	18 (9.7)
Traumatic brain injury	4 (2.2)

**Nutritional status**	
Fully dependent	26 (14.1)
Partially dependent	53 (28.6)
Just needs supervision	19 (10.3)
Independent	87 (47.0)

**Presence of feeding route modification**	42 (22.7)

**Presence of dietary modification**	146 (78.9)

Note: SD = standard deviation; min-max = minimum-maximum.

**Table 2:** Descriptive characteristics of the CHOMPS-Turkish version.

	Test			Retest			Test-retest
	Coefficient of variation	Corrected item-to-total correlation	Cronbach’s α if item deleted	Coefficient of variation	Corrected item-to-total correlation	Cronbach’s α if item deleted	Cohen’s κ coefficient
**Item 1**	0.19	0.737	0.779	0.18	0.715	0.738	1.000
**Item 2**	0.23	0.686	0.787	0.28	0.652	0.746	0.908
**Item 3**	0.22	0.645	0.790	0.27	0.606	0.749	1.000
**Item 4**	0.17	0.706	0.782	0.18	0.684	0.740	1.000
**Item 5**	0.25	0.648	0.791	0.26	0.671	0.750	0.996
**Item 6**	0.19	0.726	0.780	0.26	0.702	0.738	0.977
**Item 7**	0.23	0.613	0.791	0.27	0.669	0.751	1.000
**Item 8**	0.24	0.607	0.795	0.29	0.672	0.756	0.946
**Item 9**	0.16	0.859	0.778	0.17	0.750	0.736	1.000
**Item 10**	0.12	0.837	0.775	0.13	0.733	0.732	1.000
**Item 11**	0.15	0.822	0.776	0.14	0.796	0.735	0.971
**Item 12**	0.15	0.816	0.773	0.16	0.816	0.731	0.975
**Item 13**	0.19	0.833	0.781	0.17	0.812	0.738	1.000
**Item 14**	0.12	0.801	0.781	0.17	0.780	0.738	0.975
**Item 15**	0.15	0.823	0.775	0.16	0.704	0.732	1.000
**Item 16**	0.17	0.797	0.782	0.18	0.771	0.741	1.000
**Item 17**	0.18	0.730	0.782	0.19	0.708	0.740	1.000
**Item 18**	0.26	0.748	0.779	0.18	0.730	0.738	1.000
**Item 19**	0.12	0.738	0.777	0.19	0.715	0.735	1.000
**Item 20**	0.13	0.820	0.774	0.18	0.806	0.732	1.000
**Item 21**	0.13	0.785	0.774	0.16	0.750	0.733	0.971
**Item 22**	0.15	0.867	0.771	0.13	0.841	0.730	0.974
**Item 23**	0.14	0.769	0.771	0.12	0.745	0.730	0.977
**Item 24**	0.17	0.767	0.787	0.18	0.737	0.745	0.959
**Item 25**	0.17	0.798	0.766	0.25	0.780	0.724	0.975
**Item 26**	0.14	0.815	0.767	0.17	0.800	0.724	0.973
**Item 27**	0.15	0.852	0.765	0.19	0.816	0.723	0.975
**Item 28**	0.11	0.872	0.769	0.12	0.803	0.727	0.954
**Item 29**	0.13	0.778	0.773	0.24	0.725	0.731	0.977
**Item 30**	0.16	0.713	0.787	0.27	0.709	0.745	0.928
**Item 31**	0.17	0.722	0.785	0.18	0.696	0.744	0.891
**Item 32**	0.12	0.861	0.771	0.14	0.857	0.730	0.960
**Item 33**	0.15	0.902	0.770	0.16	0.882	0.728	0.968
**Item 34**	0.14	0.666	0.785	0.26	0.656	0.743	1.000
**Item 35**	0.10	0.853	0.775	0.10	0.839	0.733	0.965
**Item 36**	0.14	0.850	0.772	0.14	0.805	0.730	0.978
**Item 37**	0.19	0.750	0.778	0.18	0.790	0.739	0.945
**Item 38**	0.26	0.664	0.791	0.28	0.651	0.748	1.000
**Item 39**	0.16	0.780	0.776	0.15	0.741	0.734	0.970
**Item 40**	0.16	0.718	0.781	0.18	0.699	0.739	1.000
**Item 41**	0.17	0.761	0.776	0.27	0.717	0.735	0.958
**Item 42**	0.13	0.854	0.771	0.19	0.825	0.730	0.970
**Item 43**	0.15	0.874	0.767	0.17	0.874	0.724	1.000
**Item 44**	0.18	0.748	0.782	0.14	0.727	0.740	1.000
**Item 45**	0.28	0.692	0.790	0.16	0.670	0.747	1.000
**Item 46**	0.19	0.695	0.796	0.19	0.669	0.754	1.000
**Item 47**	0.15	0.718	0.787	0.16	0.751	0.746	0.907
**Item 48**	0.12	0.736	0.793	0.12	0.729	0.750	1.000
**Item 49**	0.27	0.843	0.775	0.17	0.793	0.734	1.000
**Item 50**	0.13	0.802	0.771	0.14	0.785	0.729	0.930
**Item 51**	0.15	0.718	0.792	0.28	0.692	0.749	0.873
**Item 52**	0.17	0.788	0.779	0.19	0.811	0.735	0.866
**Item 53**	0.17	0.713	0.781	0.19	0.738	0.737	0.861
**Item 54**	0.19	0.855	0.770	0.19	0.842	0.728	1.000
**Item 55**	0.25	0.755	0.781	0.14	0.744	0.739	1.000
**Item 56**	0.18	0.848	0.769	0.14	0.837	0.727	1.000
**Item 57**	0.17	0.859	0.769	0.15	0.829	0.727	0.969
**Item 58**	0.23	0.766	0.774	0.23	0.735	0.733	0.970
**Item 59**	0.12	0.810	0.769	0.19	0.776	0.728	0.972
**Item 60**	0.12	0.870	0.770	0.14	0.842	0.728	0.973
**Item 61**	0.27	0.732	0.783	0.19	0.718	0.741	1.000
**Item 62**	0.27	0.676	0.783	0.17	0.660	0.740	0.846
**Item 63**	0.25	0.786	0.772	0.27	0.770	0.730	1.000

Note: ChOMPS = Child oral and motor proficiency scale.

**Table 3:** Test–retest reliability of the ChOMPS–Turkish version.

	ICC (95% CI)	p
Complex movement patterns score	0.998 (0.997–0.999)	<0.001
Basic movement patterns score	0.997 (0.996–0.998)	<0.001
Oral-motor coordination score	0.997 (0.995–0.998)	<0.001
Fundamental oral-motor skills score	0.999 (0.999–0.999)	<0.001
Total ChOMPS-Turkish version score	0.998 (0.998–0.999)	<0.001

Note: ICC = intraclass correlation coefficient; CI = confidence interval; ChOMPS = Child oral and motor proficiency scale.

**Table 4:** Correlations between the ChOMPS–Turkish version and validity tests (n = 185).

	Pedi-EAT-10 Correlation coefficient (r)	p	FOIS Correlation coefficient (r)	p
**Complex movement patterns score**	−0.602	<0.001	0.562	<0.001
**Basic movement patterns score**	−0.859	<0.001	0.657	<0.001
**Oral-motor coordination score**	−0.613	<0.001	0.602	<0.001
**Fundamental oral-motor skills score**	−0.603	<0.001	0.605	<0.001
**Total ChOMPS-Turkish version score**	−0.682	<0.001	0.708	<0.001

Note: ChOMPS = Child oral and motor proficiency scale; FOIS = functional oral intake scale; Pedi-EAT-10 = 10-item pediatric eating assessment tool.

**Table 5:** Distribution of dysphagia status according to ChOMPS and Pedi-EAT-10 scores.

	ChOMPS – Turkish version		
	Normal feeding skills	Abnormal feeding skills	n (%)
Pedi-EAT-10 indicating no dysphagia	57 (90.5)	14 (11.5)	71 (38.4)
Pedi-EAT-10 indicating dysphagia	6 (9.5)	108 (88.5)	114 (61.6)
*n* (%)	63 (34.1)	122 (65.9)	185 (100)

Note: ChOMPS = Child oral and motor proficiency scale; min-max = minimum-maximum.

**Table 6:** ROC curve analysis results.

	95% CI (lower-upper)
Sensitivity (%)	94.74 (88.90–98.04)
Specificity (%)	80.28 (69.14–88.78)
Positive PV (%)	88.52 (82.80–92.51)
Negative PV (%)	90.48 (81.21–95.43)
Positive LR	4.80 (3.00–7.70)
Negative LR	0.07 (0.03–0.14)
AUC	0.875 (0.815–0.935)
Accuracy (%)	89.19 (83.80–93.27)

Note: CI = confidence interval; AUC = area under the ROC curve; PV = predictive value; LR = likelihood ratio.
